# A new Schiff base nickel(II) complex: {5,5′-dihydr­oxy-2,2′-[*o*-phenyl­enebis(nitrilo­methyl­idyne)]diphenolato}nickel(II) methanol disolvate

**DOI:** 10.1107/S160053680904063X

**Published:** 2009-10-13

**Authors:** Meiju Niu, Guihua Liu, Daqi Wang, Jianmin Dou

**Affiliations:** a College of Chemistry and Chemical Engineering, Liaocheng University, Shandong 252059, People’s Republic of China

## Abstract

The monomeric title nickel(II) complex of a salicylaldimine, [Ni(C_20_H_14_N_2_O_4_)]·2CH_3_OH, was obtained by the reaction of 2,4-dihydroxy­benzaldehyde and 1,2-phenyl­enediamine with nickel(II) acetate. The Ni^II^ atom is coordinated by two N atoms [Ni—N = 1.839 (2) Å] and two O atoms [Ni—O = 1.8253 (19) Å] in an approximately square-planar geometry. In the crystal structure, inter­molecular O—H⋯O hydrogen bonds link the mol­ecules into a chain along [001].

## Related literature

For related structures, see: Amirnasr *et al.* (2006 ▶); Shi *et al.* (2004 ▶); Chen *et al.* (2009 ▶); Hermindez-Molina *et al.* (1997 ▶); Zhang *et al.* (2009 ▶).
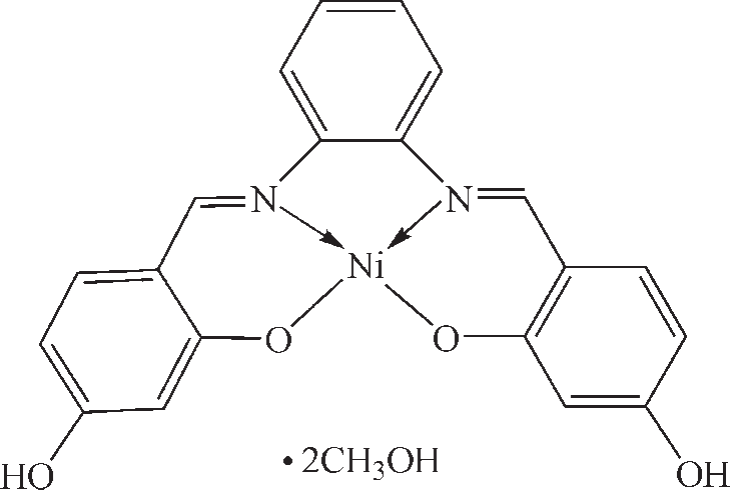


## Experimental

### 

#### Crystal data

[Ni(C_20_H_14_N_2_O_4_)]·2CH_4_O
*M* *_r_* = 469.13
Monoclinic, 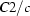

*a* = 15.673 (3) Å
*b* = 15.090 (2) Å
*c* = 8.8680 (2) Å
β = 104.593 (3)°
*V* = 2029.7 (5) Å^3^
*Z* = 4
Mo *K*α radiation
μ = 1.00 mm^−1^
*T* = 298 K
0.31 × 0.14 × 0.13 mm

#### Data collection

Siemens SMART CCD area-detector diffractometer
Absorption correction: multi-scan (*SADABS*; Sheldrick, 1996 ▶) *T*_min_ = 0.747, *T*_max_ = 0.881
5206 measured reflections
1788 independent reflections
1333 reflections with *I* > 2σ(*I*)
*R*_int_ = 0.049

#### Refinement

*R*[*F* ^2^ > 2σ(*F* ^2^)] = 0.040
*wR*(*F* ^2^) = 0.081
*S* = 1.00
1788 reflections
141 parameters
H-atom parameters constrained
Δρ_max_ = 0.35 e Å^−3^
Δρ_min_ = −0.35 e Å^−3^

### 

Data collection: *SMART* (Siemens, 1996 ▶); cell refinement: *SAINT* (Siemens, 1996 ▶); data reduction: *SAINT*; program(s) used to solve structure: *SHELXS97* (Sheldrick, 2008 ▶); program(s) used to refine structure: *SHELXL97* (Sheldrick, 2008 ▶); molecular graphics: *SHELXTL* (Sheldrick, 2008 ▶); software used to prepare material for publication: *SHELXTL*.

## Supplementary Material

Crystal structure: contains datablocks I, global. DOI: 10.1107/S160053680904063X/ds2006sup1.cif

Structure factors: contains datablocks I. DOI: 10.1107/S160053680904063X/ds2006Isup2.hkl

Additional supplementary materials:  crystallographic information; 3D view; checkCIF report

## Figures and Tables

**Table 1:** Hydrogen-bond geometry (Å, °)

*D*—H⋯*A*	*D*—H	H⋯*A*	*D*⋯*A*	*D*—H⋯*A*
O2—H2⋯O3^i^	0.82	1.97	2.734 (3)	154
O3—H3⋯O1^ii^	0.82	1.98	2.797 (3)	172

Symmetry codes: (i) 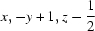; (ii) 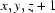.

**Fig. 1.**
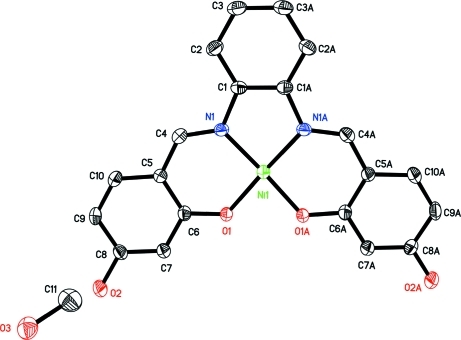
The molecular structure of the compound, with atom labels and 30% probability displacement ellipsoids for non-H atoms.

**Fig. 2.**
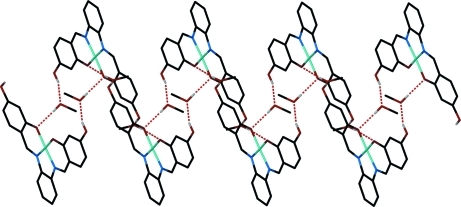
Crystal packing of the compound, showing a one-dimensional chain linked by O—H···O hydrogen bonds (dashed lines).

## supplementary crystallographic information

### Comment

Nickel complexes have attracted intensive interest in the past decade because they play important roles in bioinorganic chemistry and redox enzyme systems (Amirnasr *et al.*, 2006). In a continuation of a study of Schiff base ligands and their nickel(II) complexes, we report here the title complex (Fig. 1), in which the main plane being formed by the three phenyl and the N_2_O_2_. The angles O1—Ni1—N1A and O1A—Ni1—N1 (177.30 (10)°) indicate that the coordination geometry of the nickel atom is four-coordinate in an approximately square planar, which acts as a tetradentate ligand through its *o*-phenylenediamine N atoms and its deprotonated phenol O atoms. This square planar geometry is the most usual for Ni^II^ complexes (Shi *et al.*, 2004) in the N_2_0_2_ donor set with Schiff base ligands. The Ni—O distances of 1.8253 (19)Å are very close to the corresponding values in related structures(1.820 Å, Chen *et al.*, 2009). However, the Ni—N distances of 1.8392 (2)Å are significantly shorter than that for a related complex (1.859 Å, Hemindez-Molina *et al.*, 1997). As shown in Fig. 2, intermolecular O—H···O hydrogen bonds (Table 1) link the molecules into a one-dimensional chain along [0 0 1] direction (Zhang *et al.*, 2009).

### Experimental

*o*-Phenylenediamine(1 mmol, 108.22 mg) was dissolved in hot methanol (20 ml) and added dropwise to a methanol solution (10 ml) of 2,4-dihydroxybenzaldehyde (2 mmol, 276.2 mg). The mixture was then stirred at 323 K for 4 h. The triethylamine solution (3 ml) of nickel (II) acetate (1.5 mmol, 292.2 mg) was then added dropwise and the mixture stirred for another 4 h, at which point a red precipitate collected by suction filtration and washed with ethanol and ether. Crystals of the title compound suitable for X-ray analysis were from the methanol and dimethylsulfoxide solution after about one week.

### Refinement

All H atoms were placed geometrically and treated as riding on their parent atoms with C—H = 0.96 Å (methylene) or 0.93 Å (aromatic), 0.82 Å (hydroxyl) and *U*_iso_(H) = 1.2*U*_eq_(C).

### Crystal data

**Table:**

[Ni(C_20_H_14_N_2_O_4_)]·2CH_4_O	*F*(000) = 976
*M**_r_* = 469.13	*D*_x_ = 1.535 Mg m^−^^3^
Monoclinic, *C*2/*c*	Mo *K*α radiation, λ = 0.71073 Å
Hall symbol: -C 2yc	Cell parameters from 1287 reflections
*a* = 15.673 (3) Å	θ = 2.7–23.9°
*b* = 15.090 (2) Å	µ = 1.00 mm^−^^1^
*c* = 8.8680 (2) Å	*T* = 298 K
β = 104.593 (3)°	Block, red
*V* = 2029.7 (5) Å^3^	0.31 × 0.14 × 0.13 mm
*Z* = 4	

### Data collection

**Table:**

Siemens SMART CCD area-detector diffractometer	1788 independent reflections
Radiation source: fine-focus sealed tube	1333 reflections with *I* > 2σ(*I*)
graphite	*R*_int_ = 0.049
φ and ω scans	θ_max_ = 25.0°, θ_min_ = 1.9°
Absorption correction: multi-scan (*SADABS*; Sheldrick, 1996)	*h* = −15→18
*T*_min_ = 0.747, *T*_max_ = 0.881	*k* = −17→16
5206 measured reflections	*l* = −9→10

### Refinement

**Table:**

Refinement on *F*^2^	Primary atom site location: structure-invariant direct methods
Least-squares matrix: full	Secondary atom site location: difference Fourier map
*R*[*F*^2^ > 2σ(*F*^2^)] = 0.040	Hydrogen site location: inferred from neighbouring sites
*wR*(*F*^2^) = 0.081	H-atom parameters constrained
*S* = 1.00	*w* = 1/[σ^2^(*F*_o_^2^) + (0.0294*P*)^2^] where *P* = (*F*_o_^2^ + 2*F*_c_^2^)/3
1788 reflections	(Δ/σ)_max_ < 0.001
141 parameters	Δρ_max_ = 0.35 e Å^−^^3^
0 restraints	Δρ_min_ = −0.35 e Å^−^^3^

### Special details

**Table:**

Geometry. All e.s.d.'s (except the e.s.d. in the dihedral angle between two l.s. planes) are estimated using the full covariance matrix. The cell e.s.d.'s are taken into account individually in the estimation of e.s.d.'s in distances, angles and torsion angles; correlations between e.s.d.'s in cell parameters are only used when they are defined by crystal symmetry. An approximate (isotropic) treatment of cell e.s.d.'s is used for estimating e.s.d.'s involving l.s. planes.
Refinement. Refinement of *F*^2^ against ALL reflections. The weighted *R*-factor *wR* and goodness of fit *S* are based on *F*^2^, conventional *R*-factors *R* are based on *F*, with *F* set to zero for negative *F*^2^. The threshold expression of *F*^2^ > σ(*F*^2^) is used only for calculating *R*-factors(gt) *etc*. and is not relevant to the choice of reflections for refinement. *R*-factors based on *F*^2^ are statistically about twice as large as those based on *F*, and *R*- factors based on ALL data will be even larger.

### Fractional atomic coordinates and isotropic or equivalent isotropic displacement parameters (Å^2^)

**Table:**

	*x*	*y*	*z*	*U*_iso_*/*U*_eq_	
Ni1	0.0000	0.21153 (3)	−0.2500	0.03428 (19)	
N1	0.06329 (15)	0.12289 (14)	−0.1294 (3)	0.0322 (6)	
O1	0.06161 (13)	0.30290 (12)	−0.1384 (2)	0.0407 (5)	
O2	0.23991 (13)	0.45598 (13)	0.2855 (2)	0.0538 (6)	
H2	0.2136	0.4976	0.2348	0.081*	
O3	0.11150 (15)	0.42336 (14)	0.6598 (3)	0.0615 (7)	
H3	0.0921	0.3895	0.7154	0.092*	
C1	0.03571 (17)	0.03697 (17)	−0.1846 (3)	0.0340 (7)	
C2	0.0716 (2)	−0.04279 (19)	−0.1201 (4)	0.0438 (8)	
H2A	0.1194	−0.0433	−0.0332	0.053*	
C3	0.0357 (2)	−0.12082 (19)	−0.1861 (4)	0.0476 (9)	
H3A	0.0598	−0.1744	−0.1441	0.057*	
C4	0.12589 (19)	0.1350 (2)	−0.0019 (4)	0.0384 (8)	
H4	0.1529	0.0847	0.0494	0.046*	
C5	0.15581 (18)	0.21777 (19)	0.0639 (3)	0.0330 (7)	
C6	0.12162 (18)	0.2986 (2)	−0.0053 (3)	0.0352 (7)	
C7	0.1515 (2)	0.3782 (2)	0.0694 (4)	0.0417 (8)	
H7	0.1292	0.4315	0.0235	0.050*	
C8	0.21309 (19)	0.3788 (2)	0.2092 (4)	0.0385 (7)	
C9	0.2494 (2)	0.3000 (2)	0.2786 (4)	0.0461 (8)	
H9	0.2925	0.3007	0.3723	0.055*	
C10	0.22071 (19)	0.2219 (2)	0.2066 (4)	0.0434 (8)	
H10	0.2448	0.1693	0.2533	0.052*	
C11	0.0943 (3)	0.3892 (2)	0.5095 (5)	0.0717 (11)	
H11A	0.0431	0.4176	0.4454	0.107*	
H11B	0.0839	0.3266	0.5123	0.107*	
H11C	0.1440	0.3997	0.4671	0.107*	

### Atomic displacement parameters (Å^2^)

**Table:**

	*U*^11^	*U*^22^	*U*^33^	*U*^12^	*U*^13^	*U*^23^
Ni1	0.0358 (3)	0.0298 (3)	0.0330 (3)	0.000	0.0007 (2)	0.000
N1	0.0334 (14)	0.0260 (13)	0.0370 (15)	0.0003 (11)	0.0083 (12)	0.0010 (12)
O1	0.0474 (13)	0.0288 (11)	0.0357 (12)	−0.0001 (10)	−0.0088 (10)	0.0008 (10)
O2	0.0578 (14)	0.0451 (13)	0.0460 (15)	−0.0056 (11)	−0.0101 (11)	−0.0067 (12)
O3	0.0716 (17)	0.0518 (15)	0.0575 (17)	−0.0104 (12)	0.0094 (13)	0.0108 (13)
C1	0.0379 (18)	0.0288 (16)	0.0376 (19)	0.0023 (13)	0.0138 (13)	−0.0020 (14)
C2	0.047 (2)	0.0360 (18)	0.046 (2)	0.0054 (16)	0.0088 (16)	0.0085 (16)
C3	0.061 (2)	0.0294 (16)	0.054 (2)	0.0048 (15)	0.0172 (16)	0.0030 (15)
C4	0.0375 (18)	0.0372 (18)	0.040 (2)	0.0054 (15)	0.0081 (15)	0.0062 (16)
C5	0.0325 (16)	0.0320 (16)	0.0330 (16)	0.0017 (15)	0.0053 (13)	−0.0008 (15)
C6	0.0319 (16)	0.0385 (18)	0.0326 (17)	−0.0002 (15)	0.0035 (13)	−0.0006 (15)
C7	0.046 (2)	0.0350 (17)	0.038 (2)	−0.0007 (15)	−0.0007 (15)	−0.0017 (15)
C8	0.0373 (18)	0.0395 (18)	0.0364 (19)	−0.0043 (15)	0.0051 (15)	−0.0070 (16)
C9	0.0395 (18)	0.054 (2)	0.0352 (19)	0.0037 (17)	−0.0088 (14)	−0.0008 (17)
C10	0.0420 (19)	0.0410 (19)	0.0409 (19)	0.0068 (16)	−0.0014 (15)	0.0055 (17)
C11	0.081 (3)	0.064 (3)	0.072 (3)	−0.010 (2)	0.025 (2)	0.000 (2)

### Geometric parameters (Å, °)

**Table:**

Ni1—O1	1.8253 (19)	C3—H3A	0.9300
Ni1—O1^i^	1.8253 (19)	C4—C5	1.408 (4)
Ni1—N1^i^	1.839 (2)	C4—H4	0.9300
Ni1—N1	1.839 (2)	C5—C6	1.409 (4)
N1—C4	1.310 (4)	C5—C10	1.411 (4)
N1—C1	1.414 (3)	C6—C7	1.395 (4)
O1—C6	1.312 (3)	C7—C8	1.366 (4)
O2—C8	1.360 (3)	C7—H7	0.9300
O2—H2	0.8200	C8—C9	1.393 (4)
O3—C11	1.391 (4)	C9—C10	1.361 (4)
O3—H3	0.8200	C9—H9	0.9300
C1—C2	1.389 (3)	C10—H10	0.9300
C1—C1^i^	1.394 (6)	C11—H11A	0.9600
C2—C3	1.371 (4)	C11—H11B	0.9600
C2—H2A	0.9300	C11—H11C	0.9600
C3—C3^i^	1.377 (6)		
			
O1—Ni1—O1^i^	81.88 (12)	C4—C5—C6	122.5 (3)
O1—Ni1—N1^i^	177.30 (10)	C4—C5—C10	120.1 (3)
O1^i^—Ni1—N1^i^	95.74 (9)	C6—C5—C10	117.5 (3)
O1—Ni1—N1	95.74 (9)	O1—C6—C7	117.6 (3)
O1^i^—Ni1—N1	177.30 (10)	O1—C6—C5	122.9 (3)
N1^i^—Ni1—N1	86.67 (15)	C7—C6—C5	119.6 (3)
C4—N1—C1	121.6 (3)	C8—C7—C6	120.8 (3)
C4—N1—Ni1	125.3 (2)	C8—C7—H7	119.6
C1—N1—Ni1	113.11 (19)	C6—C7—H7	119.6
C6—O1—Ni1	127.69 (19)	O2—C8—C7	121.1 (3)
C8—O2—H2	109.5	O2—C8—C9	118.1 (3)
C11—O3—H3	109.5	C7—C8—C9	120.8 (3)
C2—C1—C1^i^	119.95 (18)	C10—C9—C8	118.8 (3)
C2—C1—N1	126.5 (3)	C10—C9—H9	120.6
C1^i^—C1—N1	113.53 (15)	C8—C9—H9	120.6
C3—C2—C1	119.2 (3)	C9—C10—C5	122.5 (3)
C3—C2—H2A	120.4	C9—C10—H10	118.7
C1—C2—H2A	120.4	C5—C10—H10	118.7
C2—C3—C3^i^	120.81 (18)	O3—C11—H11A	109.5
C2—C3—H3A	119.6	O3—C11—H11B	109.5
C3^i^—C3—H3A	119.6	H11A—C11—H11B	109.5
N1—C4—C5	125.5 (3)	O3—C11—H11C	109.5
N1—C4—H4	117.2	H11A—C11—H11C	109.5
C5—C4—H4	117.2	H11B—C11—H11C	109.5

Symmetry codes: (i) −*x*, *y*, −*z*−1/2.

### Hydrogen-bond geometry (Å, °)

**Table:**

*D*—H···*A*	*D*—H	H···*A*	*D*···*A*	*D*—H···*A*
O2—H2···O3^ii^	0.82	1.97	2.734 (3)	154
O3—H3···O1^iii^	0.82	1.98	2.797 (3)	172

Symmetry codes: (ii) *x*, −*y*+1, *z*−1/2; (iii) *x*, *y*, *z*+1.
